# Pentraxin-3, Interleukin-6, and Acute Appendicitis: Biomarkers That Need Further Exploration

**DOI:** 10.7759/cureus.9991

**Published:** 2020-08-24

**Authors:** Ayman Abouhamda

**Affiliations:** 1 Independent Researcher, National Coalition of Independent Scholars, Jeddah, SAU

**Keywords:** acute appendicitis, pentraxin-3, interleukin-6, pediatric surgery, diagnosis, alvarado score, accurate diagnosis

## Abstract

Acute appendicitis is one of the most common abdominal emergencies that present in the hospital. With acute appendicitis, there is always a possibility of complications of perforation and peritonitis. Even though medicine has advanced substantially in different investigational modalities, appendicitis is confirmed clinically with the aid of a clinical approach (i.e., Alvarado Score), laboratory, and investigational modalities. However, biomarkers such as pentraxin-3 and interleukin-6 have been recently researched to assess the possibility of confirming the diagnosis of acute appendicitis in both adults and the pediatric age group. This article breaks down the previous research on pentraxin-3 and interleukin-6 biomarkers in relation to appendicitis and proposes a new hypothetical way of confirming the diagnosis.

## Introduction and background

The pentraxin superfamily consists of two main arms, a short arm that includes well-known established proteins known as the C-reactive protein (CRP) and serum amyloid P, whereas pentraxin-3 (PTX3), the acute phase protein, was identified in 1990 as the long arm of the pentraxin family being expressed in various types of tissues and cells in response to inflammation, such as monocytes/macrophages, dendritic cells, endothelial cells, smooth muscle cells, vascular endothelial cells, and fibroblasts [1]. However, they are not produced by hepatocytes, which are the major production site of CRP. They are instead produced locally at the inflammatory site [2-4]. Similar to the CRP, studies have recently demonstrated the role of PTX3 in humoral innate immunity, where it has the ability to bind to the apoptotic cells as well as induce macrophage secretion of the immunosuppressive cytokines [2,5]. This great range of inflammatory response is expressed in both acute and chronic types of inflammation on a local and systemic level in the human body [1]. Furthermore, PTX3, which has a value of <2 ng/mL in healthy individuals [6], has the power to interact with C1q, the first component of the classical component cascade [1], which is initiated by both immunoglobulin (Ig) M and IgG [7]. Moreover, PTX3 has been found in inflammatory areas concerning various types of cytokines, such as interleukin (IL)-6, IL-8, IL-10, and procalcitonin [8,9]. Such pathways are found in all kinds of infections, immune disease, cardiac disease, muscle fibers, septic patients, and even wound healing [1,3,5,10].

## Review

Pentraxin-3 and appendicitis

Acute appendicitis is the most common and frequent abdominal complaint with a 7% occurrence rate and challenges in diagnosis despite improvement in medicine [11,12]. It is clinically diagnosed rigorously by the Alvarado Score with the aid of laboratory investigations, ultrasound, and computed tomography (CT) scans to intervene to prevent perforation and serious complications. On a pathophysiological level, appendicitis occurs as the lumen becomes obstructed, which leads to local inflammation, where it can spread to the surrounding peritoneum and pericaecal fat [11]. As a result of such obstruction, high luminal pressure can subsequently lead to vascular congestion and further worsen the local inflammation by the gangrenous effect of the obstructed blood and lymph flow. This makes it a perfect medium for bacterial growth and colonization, and, in some cases, complications such as perforation and peritonitis occur [11]. As IL-6 has been reported to induce the acute phase response caused by bacterial endotoxemia. Zviedre et al. reported that IL-6 can provide more sensitivity in acute appendicitis when used in parallel with a white blood count (WBC) to improve the sensitivity of a diagnosis of appendicitis [13]. However, PTX3 has been linked to various cytokines as an inflammatory response related to IL-6. Therefore, it can shed further light on becoming a biomarker modality in diagnosing appendicitis or it can play a role in increasing the sensitivity rate if used in concurrence with the Alvarado Score as well as other diagnostic modalities.

To assess the level of research conducted on such a topic, an approach similar to that of a systematic review was used. A systematic electronic database search was conducted for relevant studies published without any restriction on the year of publishing in the following seven databases using keywords and medical subject (MeSH) terms: Google Scholar, Scopus, Web of Science (Institute for Scientific Information), PubMed, Cochrane Central Register of Controlled Trials (CENTRAL), Embase, and CINAHL. A search using a combination of all possible word terms with “Pentraxin-3” AND “Appendicitis” in the title, abstract, and all fields was conducted. A total of four papers published between 2017 and 2020 were found, as well as an additional paper appraising one of the four published manuscripts.

Aygun et al. in 2017 explained the first reported research studying the association between PTX3 levels and acute appendicitis and targeted patients who were 17 years of age and older and divided them into five groups. Meanwhile, all cases that were found to have negative post-operative pathology for acute appendicitis were excluded. They reported that PTX3 was as sensitive and specific as CRP, with elevated levels of PTX3 being observed in those with abdominal pain associated with an inflammatory pathology. However, they concluded that PTX3 alone is not sufficient to make a diagnosis without the aid of other investigations [4].

The second study was a prospective study in 2019 on a pediatric group of patients who were 18 years and younger. A total of 88 patients were included in the study and were divided into three groups: volunteers (n=28), non-specific abdominal pain group while kept under observation (n=28), and those who underwent appendectomy (n=34). Oztan et al. compared levels of PTX3, WBC, absolute neutrophil count, neutrophil/lymphocyte ratio, and CRP. Similar to the previous study, high PTX3 sensitivity and specificity were found when compared to the other biomarkers tested. PTX3 showed superiority and accuracy in the third group [14]. However, the median PTX3 level reported in this study was 12.60 ng/mL, which is much higher than the median reported by Aygun et al. [4].

The next study testing the PTX3 role in appendicitis was conducted in 2019 on 172 adult patients divided into three groups. The first group was a control group (n=65), which presented in the clinic with a hernia and was invited to the study. The second group (n=84) had a diagnosis of non-perforated acute appendicitis, whereas the last group (n=28) consisted of perforated acute appendicitis. Interestingly, PTX3 levels were high in the non-perforated group and even higher in the perforated one, with median PTX3 values of 7.09 ng/mL and 13.89 ng/mL, respectively. Furthermore, IL-6 levels were also found higher in the perforated group, with a median value of 532.2 pg/mL, and in the non-perforated acute appendicitis group, with a median value of 250.8 pg/mL. Regarding specificity and sensitivity, PTX3 reported a sensitivity of 95% and a specificity of 100%, whereas IL-6 showed a sensitivity of 93.8% and a specificity of 84.6%. This was the only study that assessed the clinical likelihood of the probability of perforation using the AUC (area under the curve) and ROC (receiver operating characteristics) curve on PTX3, with an 81.3% chance for cases with appendicitis to progress to perforation, with a cut-off point of higher than 9.56 ng/mL. The IL-6 prediction probability was 75%, with a cut-off value of 492 pg/mL. Meanwhile, the overall accuracy of confirming the diagnosis of acute appendicitis using PTX3 and IL-6 was 97.2% and 90.4%, respectively [6].

The final study to assess PTX3 was conducted on a pediatric group of 55 patients under the age of 18 years. In a similar pattern, patients were also divided into healthy, non-perforated, and perforated appendicitis. The median PTX3 value for normal individuals was 1.01 ng/mL, and the median value before surgery was reported as 20.68 ng/mL. However, according to Ates et al., the cut-off value of PTX3 in the pediatrics group for those with appendicitis was 1.30 ng/mL, with a sensitivity of 75% and a specificity of 100%. Lastly, the reported positive and negative predictive values according to the ROC analysis were 100% and 60%, respectively [15].

Future research implementation

**Figure 1:**
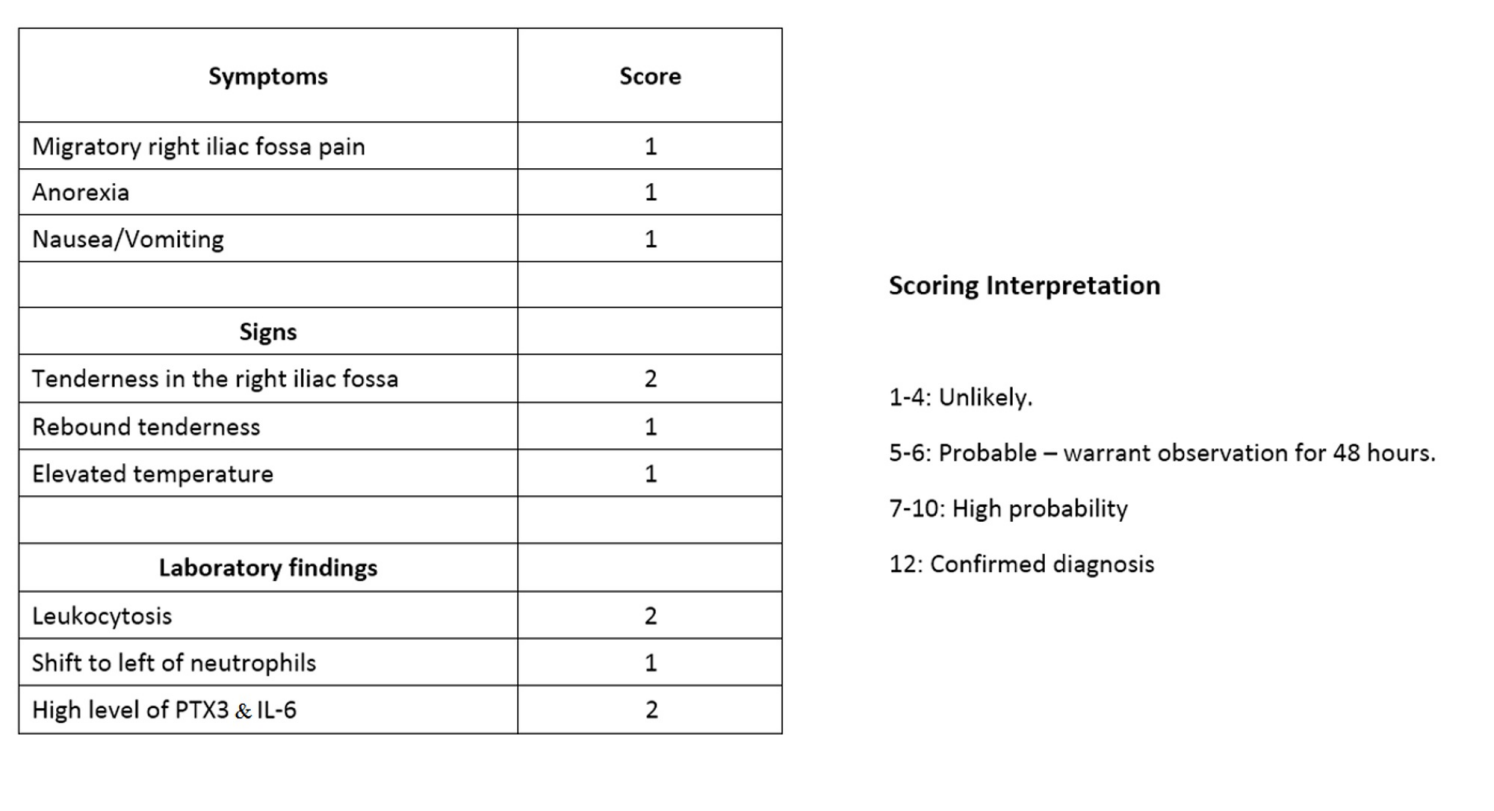
Hypothetical module of a modified Alvarado Score

At present, acute appendicitis is presenting a challenge in diagnosis, and clinicians are still relying heavily on the clinical diagnosis using the Alvarado Score along with investigation and laboratory work to try to confirm the diagnosis in such cases. However, the previous studies show how PTX3 with the aid of IL-6 might change that in the near future by not only diagnosing acute appendicitis but also predicting the likelihood of perforation in those individuals. One important limitation of the four studies is that they were single-center studies with different approaches [4,6,14,15]. Also, only two of them assessed the association of IL-6 and PTX3 with appendicitis. Furthermore, it can be difficult to confirm a diagnosis of appendicitis with biomarkers that are usually raised due to various kinds of inflammations and infections that occur in the body [9,16]. Perhaps, proposing a new diagnostic hypothetical module of the Alvarado Score [17] in children would help to confirm the diagnosis and prevent the risk of a negative diagnosis of appendicitis. Testing this hypothetical module on a multi-center level might give us a new perspective on the importance of exploring this biomarker and help save more lives (Figure 1).

## Conclusions

Acute appendicitis remains one of the most common abdominal pathologies in emergency surgical care. Even though many investigational modalities are available, it is still susceptible to misdiagnosis without a way to completely confirm the diagnosis and without putting the patient through many radiological investigational efforts. Therefore, further studies on a multicenter level are needed to explore the role of PTX3 and IL-6 in acute appendicitis alongside the Alvarado Score.
